# The Primary Immunodeficiency Database in Japan

**DOI:** 10.3389/fimmu.2021.805766

**Published:** 2022-01-10

**Authors:** Kanako Mitsui-Sekinaka, Yujin Sekinaka, Akifumi Endo, Kohsuke Imai, Shigeaki Nonoyama

**Affiliations:** ^1^ Department of Pediatrics, National Defense Medical College, Saitama, Japan; ^2^ Department of Pediatrics and Clinical Research Center, Tokyo Medical and Dental University, Tokyo, Japan; ^3^ Department of Community Pediatrics, Perinatal and Maternal Medicine, Graduate School of Medical and Dental Sciences, Tokyo Medical and Dental University, Tokyo, Japan

**Keywords:** primary immunodeficiency, Primary Immunodeficiency Database in Japan, Japanese Society for Immunodeficiency and Autoinflammatory Diseases, consultation, genetic analysis, pathogenesis

## Abstract

The Primary Immunodeficiency Database in Japan (PIDJ) is a registry of primary immunodeficiency diseases (PIDs) that was established in 2007. The database is a joint research project with research groups associated with the Ministry of Health, Labor and Welfare; the RIKEN Research Center for Allergy and Immunology (RCAI); and the Kazusa DNA Research Institute (KDRI). The PIDJ contains patient details, including the age, sex, clinical and laboratory findings, types of infections, genetic analysis results, and treatments administered. In addition, web-based case consultation is also provided. The PIDJ serves as a database for patients with PIDs and as a patient consultation service connecting general physicians with PID specialists and specialized hospitals. Thus, the database contributes to investigations related to disease pathogenesis and the early diagnosis and treatment of patients with PIDs. In the 9 years since the launch of PIDJ, 4,481 patients have been enrolled, of whom 64% have been subjected to genetic analysis. In 2017, the Japanese Society for Immunodeficiency and Autoinflammatory Diseases (JSIAD) was established to advance the diagnosis, treatment, and research in the field of PIDs and autoinflammatory diseases (AIDs). JSIAD promotes the analysis of the pathogenesis of PIDs and AIDs, enabling improved patient care and networking *via* the expansion of the database and construction of a biobank obtained from the PIDJ. The PIDJ was upgraded to “PIDJ ver.2” in 2019 by JSIAD. Currently, PIDJ ver.2 is used as a platform for epidemiological studies, genetic analysis, and pathogenesis evaluation for PIDs and AIDs.

## Introduction

Primary immunodeficiency diseases (PIDs) are rare and genetically heterogeneous disorders that impair the immune system. Recent studies have indicated the prevalence of >400 causative genes, and genetic analysis plays an important role in confirming the PID diagnosis and the selection of treatment options, which include the use of hematopoietic stem cell transplantations, gene therapy, and biological agents. Because PIDs are rare diseases, the compilation of patient details, including genetic analysis and clinical information, can contribute significantly toward evaluating the pathogenesis involved and the establishment of optimal treatment methods. Given this background, a registry of patients with primary immunodeficiency (PIDJ: Primary Immunodeficiency Database in Japan) was established in 2007, enabling an overview of Japanese patients with PIDs.

### Before the PIDJ Project

Before the launch of the PIDJ project, Japan had a retrospective, paper-based registration system. The first nationwide survey related to PIDs in Japan was performed in 1979, which was supported by the Japan Ministry of Health, in which 497 patients were registered (1). During registration, the survey included the numbers of each type of PIDs, patient age at the time of diagnosis, patient status at the time of registration, familial incidence of PIDs, and any associated complications. However, molecular analysis and sample stocking were not performed in that survey. When the causative gene associated with each patient with PIDs was identified, it was registered in each institution’s private database, as a nationwide database had not been established.

### The PIDJ Project (2007~2017)

PIDs are rare diseases, with low numbers of patients but highly variable symptoms, severity, and complications, making diagnosis by non-specialists difficult. The disease can be fatal if the diagnosis is delayed or if no appropriate therapeutic intervention is performed; therefore, consultations with PID expert doctors are required. Therefore, in 2007, we launched a website for PIDJ at the RIKEN Research Center for Allergy and Immunology (RCAI) to facilitate consultations for PID patients with local physicians or expert doctors from 13 universities across Japan. In the PIDJ network, general physicians evaluating potential PID patients consult expert doctors with experience in PID diagnosis and register for PIDJ with informed consent from the patients. The clinical information of the patients is added *via* the internet, and PID experts advise general physicians consulting with the patient. Where further analyses are required, patients’ samples are sent to RCAI or PID expert doctors to perform immunological analysis, including FACS and genetic and functional evaluations, and patient samples are preserved for future use. Genetic analysis is performed at the Kazusa DNA Research Institute (KDRI). The important goals are to enable accurate diagnosis, recommend appropriate treatments, and provide support to connect PID patients to specialized medical institutions for prompt and appropriate medical care. Thus, consultation, registration, sample stocking, molecular diagnosis, functional analysis, and advice from PID expert doctors can easily be achieved through the PIDJ network.

In the 9 years (from 2007 to 2017) since the launch of PIDJ, 4,481 patients have been registered. Genetic analysis has been performed in 2,869 patients (64% of all registered patients), and the causative gene was identified in 804 cases. The most common diagnosis was autoinflammatory disorders (39%), followed by predominantly antibody deficiencies (14.4%), combined immunodeficiencies with syndromic features (8.7%), diseases of immune dysregulation (8.7%), congenital defects of phagocytes (7.9%), and combined immunodeficiencies (3.9%) (**Table 1**).

**Table 1-1:** The total numbers of registered patients (2008~2016).

IUIS classification	Number of Patients	(%)
Combined immunodeficiencies	170	(3.9%)
Combined immunodeficiencies with syndromic features	377	(8.7%)
Predominantly antibody deficiencies	624	(14.4%)
Diseases of immune dysregulation	375	(8.7%)
Congenital defects of phagocytes	343	(7.9%)
Defects in intrinsic and innate immunity	141	(3.2%)
Autoinflammatory disorders	1692	(39%)
Complement deficiencies	24	(5.5%)
Phenocopies of PID	410	(9.5%)
Others	151	(3.5%)
Total	4307

**Table 1-2:** The total numbers of genetically diagnosed patients (2008~2016).

IUIS Classification	Genetic Defect	Number of Patients	(%)
Combined immunodeficiencies	*IL2RG*	51	(6.3%)
	*CD40L*	36	(4.4%)
	*RAG1*	13	(1.6%)
Combined immunodeficiencies with syndromic features	*WAS*	82	(10.1%)
	*STAT3*	31	(3.8%)
	*ATM*	28	(3.4%)
	*IKBKG*	16	(1.9%)
Predominantly antibody deficiencies	*BTK*	69	(8.5%)
Diseases of immune dysregulation	*XIAP*	15	(1.8%)
	*UNC13D*	15	(1.8%)
	*CTLA4*	14	(1.7%)
	*PRF1*	13	(1.6%)
Congenital defects of phagocytes	*CYBB*	54	(6.7%)
	*NCF2*	20	(2.4%)
	*ELANE*	11	(1.3%)
Defects in intrinsic and innate immunity	*STAT1*	12	(1.4%)
Autoinflammatory disorders	*MEFV*	104	(12.9%)
	*NLRP3*	15	(1.8%)
	*TNFAIP3*	13	(1.6%)
Total	89 genes	804	

The causative genes identified in more than 10 patients are listed in the Table.

Moreover, PIDJ has contributed to the identification of novel causative genes associated with PIDs. For patients in whom no mutations in known PID-causing genes are detected, cases with similar clinical symptoms and laboratory findings are selected for detailed analysis to identify novel causative genes. We have identified several causative genes of PIDs by using PIDJ database (2–13).

### PIDJ ver.2, Now and Beyond

In 2017, the Japanese Society for Immunodeficiency and Autoinflammatory Diseases (JSIAD) was established to advance the diagnosis, treatment, and research in the field of PIDs and autoinflammatory diseases (AIDs). The PIDJ was upgraded to “PIDJ ver.2” in 2019 by JSIAD. With the expansion of the database and construction of a biobank, PIDJ ver.2 is being used as a platform for epidemiological studies, genetic analysis, and pathogenesis evaluation for PIDs and AIDs. Consultation, registration, sample stocking, molecular diagnosis, functional analysis, and advice from PID expert doctors are being provided using PIDJ ver.2. The PIDJ committees are organized in JSIAD to respond to consultations from general physicians. Genetic analysis (by NGS) and sample stocking are performed at the KDRI. The JSIAD collaborating facilities (81 facilities as of June 2020) participate in PIDJ ver.2 as joint research facilities and play a role in the diagnosis and treatment of PIDs and AIDs, as well as research and education about the topic. Further, on the basis of the IUIS classification (14, 15), JSIAD has developed a panel of genes recommended for genetic testing for each PID and AID related to clinical usefulness and validity (**Table 2**).

**Table 2:** Target genes responsible for PIDs and AIDs.

IUIS Classification (2019)	Name of Panel Set	Target Genes
Table 1	Combined Immunodeficiency (1)	IL2RG, JAK3, IL7R, RAG1, RAG2, DCLRE1C, ADA, PNP, ZAP70, LIG4, NHEJ1, TBX1
	Combined Immunodeficiency (2)	AK2, CORO1A, FOXN1, PRKDC, PTPRC, STAT5B, ORAI1, STIM1, MAGT1, RAC2, CHD7, SEMA3E, POLE, ATM, CD3D,CD3E, CD247, LAT
	MHC deficiency	TAP1, TAP2, B2M, CIITA, RFXANK, RFX5, RFXAP
Table 2	Wiskott–Aldrich syndrome	WAS, ARPC1B, CDC42, WIPF1
	Hyper IgE syndromes	STAT3, TYK2, IL6R, ZNF341, ERBIN, TGFBR1,TGFBR2, SPINK5,PGM3, CARD11, DOCK8
	Immuno-osseous dysplasias	SMARCAL1, RNU4ATAC, EXTL3
	DNA mismatch-repair deficiency	ATM, MRE11, NBN, RAD50, LIG4, NHEJ1, DCLRE1C, PRKDC, DNMT3B, ZBTB24, CDCA7, HELLS, RNF168, MCM4, BLM
	Anhidrotic ectodermodysplasia with immunodeficiency	IKBKG, NFKBIA, IKBKB, ORAI1
Table 3	Profoundly decreased or absent B cells	BTK, IGHM, IGLL1, CD79A, BLNK, PIK3CD, PIK3R1, TCF3, SLC39A7, TRNT1, IKZF1, IKZF3
	Hyper-IgM syndromes	CD40LG, AICDA, CD40, UNG, INO80, PIK3CD, PIK3R1, PTEN, IKBKG
	Common variable immunodeficiency (CVID) (1)	TNFSF12, TNFSF13, TNFRSF13B, TNFRSF13C, CD19, CR2, PLCG2, IKZF1, IKZF3, NFKB1, NFKB2, SEC61A1, IRF2BP2, ATP6AP1, ARHGEF1, SH3KBP1, DNMT3B, ZBTB24, CDCA7, HELLS
	Common variable immunodeficiency (CVID) (2)	ICOS, PLCG2, LRBA, CTLA4, IL21R, MALT1, MSN, CARD11,BCL10, ITK, PIK3CD, PIK3R1, NFKB1, NFKB2
Table 4	Familial hemophagocytic lymphohistiocytosis (FHL syndromes)	PRF1, UNC13D, STX11, STXBP2, FAAP24, SLC7A7, LYST,RAB27A, AP3B1, AP3D1, SH2D1A, XIAP
	Autoimmune lymphoproliferative syndrome	FAS, FASLG, CASP8, CASP10, NRAS, KRAS, AIRE, FOXP3, IL2RA, CTLA4, LRBA, STAT3, SH2D1A, IKZF1, PIK3CD, PIK3R1, PRKCD, TNFAIP3
	IPEX syndromes	FOXP3, IL2RA, IL2RB, CTLA4, LRBA, STAT3, FERMT1, STAT1, STAT5B
	Immune dysregulation with colitis	IL10, IL10RA, IL10RB, NFAT5, TGFB1, RIPK1, FOXP3, IL2RA, CTLA4, LRBA, WAS, XIAP, CYBA, CYBB, NCF2, NCF4, TNFAIP3
	Susceptibility to EBV and lymphoproliferative conditions	SH2D1A, XIAP, CD27, RASGRP1, CARMIL2, MAGT1, PRKCD, STK4, ITK, ZAP70, MCM4, PIK3CD, PIK3R1, NFKB1, CTLA4, PRF1, STXBP2, FAS
Table 5	Congenital neutropenias (1)	ELANE, HAX1, WAS, CSF3R, SRP54, CXCR4
	Congenital neutropenias (2)	GFI1, G6PC3, SLC37A4, TAZ, VPS13B, USB1, JAGN1, CLPB
	Shwachman–Diamond syndrome	SBDS
	Leukocyte adhesion deficiency	ITGB2, SLC35C1, FERMT3, RASGRP2
	Chronic granulomatous disease (CGD)	CYBB, CYBA, NCF2, NCF4, G6PD
	Congenital defects of phagocyte	RAC2, ACTB, FPR1, CTSC, WDR1, MRTFA, SLC11A1, CEBPE, G6PD, MPO
	Familial defects of dendritic cells	GATA2, CSF2RA, CSF2RB, IRF7, IRF8
IUIS classification (2019)	Name of panel set	Target genes
Table 6	Mendelian susceptibility to mycobacterial disease (MSMD)	IL12RB1, IL12B, IL12RB2, IL23R, IFNGRl, IFNGR2, STAT1,CYBB, IRF8, TYK2, RORC, JAK1, IKBKG, GATA2
	TLR signaling pathway deficiency with bacterial susceptibility	IRAK4, MYD88, TIRAP, IKBKG, NFKBIA, IKBKB, RPSA, NKX2-5, RBCK1
	Chronic mucocutaneous candidiasis; CMC	IL17RA, IL17F, STAT1, TRAF3IP2, RORC, AIRE, STAT3, IL12RB1, IL12B, CARD9
	Predisposition to severe viral infection	STAT1, STAT2, IRF7, IFNAR1, FCGR3A, IFIH1, TLR3, TBK1,DBR1, IRF8, MCM4, TMC6, TMC8, CXCR4
Table 7	Autoinflammatory disorders	ADA2, NLRC4, TNFAIP3
	Aicardi–Goutieres syndrome (AGS)	TREX1, RNASEH2B, RNASEH2C, RNASEH2A, SAMHD1, ADAR, IFIH1
	Cryopyrin-associated periodic fever syndrome	NLRP3
	Hyper-IgD syndrome	MVK
	Pyogenic sterile arthritis, pyoderma gangrenosum, acne (PAPA) syndrome	PSTPIP1
	TNF receptor-associated periodic syndrome (TRAPS)	TNFRSF1A
	Nakajo–Nishimura syndrome	PSMB8
	Familial Mediterranean fever	MEFV
	Blau syndrome	NOD2
Table 8	Complement deficiencies	C1QA, C1QB, C1QC, C1R, C1S, C2, C3, C5, C6, C7, C8A, C8B, C9, CFB, CFI, CFP, MASP2, MBL2
	Complement deficiencies (hereditary angioedema)	SERPING1, F12, ANGPT1, PLG, CD55, CD59
Table 9	Dyskeratosis congenita	DKC1, TERC, TERT, TINF2, RTEL1, ACD, WRAP53, PARN, CTC1, DCLRE1C

## Discussion

PIDs include over 400 diseases caused by mutations in single genes, which makes them difficult to diagnose by general physicians. Because PIDs are rare diseases, patient registration is important for the diagnosis, genetic and functional analysis, and improved patient care. PIDJ was initially associated with 13 medical colleges, the RCAI, and KDRI, back in 2007. PIDJ is a nationwide network that includes patient consultations, registration, sample stocking, genetic diagnosis, molecular analysis, and advice by PID expert doctors.

A total of 4,481 patients have been registered in PIDJ, and genetic analysis has been performed in 2,869 patients (64% of all registered patients), and the causative gene was identified in 804 cases. Comparing our results with reports from registries in other countries (e.g., Europe, the United States, the Middle East, and Asia), PIDJ is characterized by a large number of registered AID patients (especially with *MEFV* mutations) (16–28). On the other hand, with regard to PIDs, adult cases that had already been diagnosed and cases that have been followed only with γ-globulin administration may not have been registered in PIDJ. This is the limitation of this registration, and future efforts should be made to ensure that all PID patients in Japan will be registered in PIDJ. We are conducting a survey of all PIDs patients in Japan to identify missing cases in the registration and are working on registering all the PIDs patients for PIDJ (29, 30).

PIDJ was upgraded to PIDJ ver.2 with the establishment of JSIAD in 2017 and has been expanded further, with additional functions to date. The PIDJ network will help doctors and researchers perform various analyses, improve disease prognosis, and advance understanding of the human immune system.

## Author Contributions

KM-S did the conception and design. KM-S, KI, and AE analyzed the data. KM-S and KI wrote the manuscript. SN, YS, and KI provided critical discussion, supervised the study, and edited the manuscript. All authors reviewed the paper. All authors contributed to the article and approved the submitted version.

## Funding

This work was supported in part by the Ministry of Health, Labour and Welfare, Japan (20316700, 20317089), Japan Society for the Promotion of Science (JSPS) KAKENHI (20K16942, 20K22916), the Ministry of Education, Culture, Sports, Technology, Japan (MEXT) KAKENHI (20288564), the Practical Research for Rare/Intractable Diseases from AMED (20314514, 20314940), and the Project for Baby and Infant Research of Health and Development to Adolescent and Young Adult from AMED (19188706).

## Conflict of Interest

The authors declare that the research was conducted in the absence of any commercial or financial relationships that could be construed as a potential conflict of interest.

## Publisher’s Note

All claims expressed in this article are solely those of the authors and do not necessarily represent those of their affiliated organizations, or those of the publisher, the editors and the reviewers. Any product that may be evaluated in this article, or claim that may be made by its manufacturer, is not guaranteed or endorsed by the publisher.
